# A 92-Year-Old Man with Abdominal Pain Following Intractable Vomiting; a Photo Quiz

**Published:** 2019-08-06

**Authors:** Chin-Chu Wu, Aming Chor-Ming Lin

**Affiliations:** 1 Department of Diagnostic Radiology, Shin Kong Wu Ho-Su Memorial Hospital, Taipei, Taiwan. ORCID: 0000-0003-2658-5775; 2 Emergency Department, Shin Kong Wu Ho-Su Memorial Hospital, Taipei, Taiwan.; 3 School of Medicine, Fu-Jen Catholic University, New Taipei city, Taiwan.

## Case presentation:

**Figure 1:**
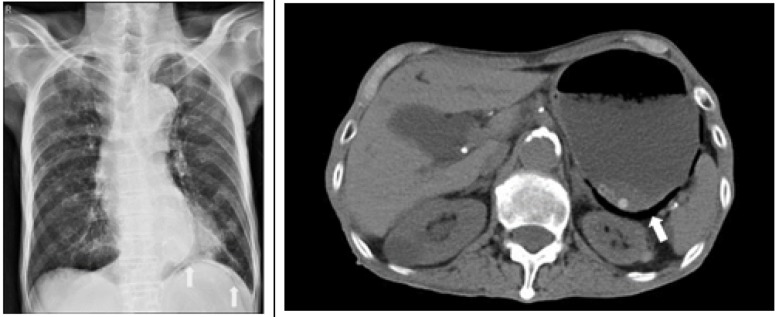
Posterior-anterior chest X-ray (left) and coronal view of abdominal computed tomography scan (right) of patient

A 92-year-old man with hypertension, chronic obstructive pulmonary disease (COPD), peptic ulcer disease and dementia presented to the emergency department with a 2-day history of abdominal pain in the left upper quadrant, distention, dry cough and intractable vomiting. On physical examination, the patient had epigastric tenderness and bowel sounds were reduced. The patient’s vital signs included blood pressure of 168/84 mmHg, heart rate of 99 beats/minutes, respiratory rate of 24 beats/minutes, and oxygen saturation of 95% in room air. His temperature was 37.9 °C. The rest of physical examination findings were unremarkable. Complete blood cell count showed the following results: leukocyte count 10100/mm3 with 85% segmented neutrophils, hemoglobin 12 g/dl, platelet 350000/microliter, and international normalized ratio (INR) of 0.97. Other laboratory findings included: glucose 167 mg/dl, blood urea nitrogen (BUN) 28 mg/dl, serum creatinine 2.0 mg/dl, sodium 135 mEq/L, potassium 3.3 mEq/L, serum glutamic oxaloacetic transaminase (SGOT) 15 U/L, total bilirubin 0.5 mg/dl, and lipase 25 U/L. The patient underwent chest X-ray and abdominal computed tomography (CT) scan without contrast material, the results of which are shown in Figure 1 and Figure 2.

**What is your diagnosis?**

**Diagnosis:**

Chest X-ray showed left lower lung interstitial opacities and air bubble below the left hemi diaphragm (Figure 1 (left), arrows). Abdominal CT scan revealed intramural gas within a dilated stomach (Figure 1 (right), arrow). A diagnosis of gastric emphysema was made. 

**Case fate:**

The patient was not toxically ill and recovered with conservative management of nasogastric decompression, intravenous fluids, total parenteral nutrition and intravenous antibiotics. The patient was discharged from the hospital 7 days after presentation. 

## Discussion

Gastric emphysema is characterized by the presence of air within the wall of the stomach. Gas within the stomach wall is an alarming finding on imaging. Early recognition is essential. The gas may be caused by gastric pneumatosis, also known as gastric emphysema or emphysematous gastritis (1). It is important to differentiate the causes of gastric pneumatosis. Gastric emphysema is usually asymptomatic and relatively benign, and resolves spontaneously with conservative treatment with bowel rest, parenteral nutrition and wide spectrum antibiotics. Gastric emphysema is a rare condition in which gas accumulates within the wall of the stomach. Gastric distension and frequent vomiting precede the formation of the intramural air. Pulmonary disease, instrumentation of the stomach, and obstructing lesions of the antrum and pylorus are also common contributing factors. 

Emphysematous gastritis is a lethal condition with high mortality (2, 3). Surgery is indicated in emphysematous gastritis because of gastric infarction, perforation or failed medical management. Emphysematous gastritis is characterized by the presence of gas in the wall of the stomach and is a severe form of phlegmonous gastritis, which develops due to invasion by gas-forming microorganisms. Air within the gastric wall along with portal venous air, sepsis and evidence of infection support the diagnosis of emphysematous gastritis (1, 2). The radiographic finding of gastric emphysema with hepatic portal venous gas is classically an ominous sign, associated with a high mortality rate. Diabetes mellitus, immunosuppression, alcohol abuse, ulcerative colitis and use of non-steroidal anti-inflammatory drugs (NSAID) have all been reported as predisposing factors (4, 5). The most commonly involved microorganisms are streptococci, Escherichia coli, Pseudomonas aeruginosa, Clostridium perforins and Staphylococcus aureus. Abdominal CT scan is the diagnostic tool of choice to detect gastric pneumatosis and helps in differentiating it with gastric emphysema or emphysematous gastritis. 

## Conclusion:

The presence of gas within the stomach wall may be associated with a wide range of conditions, ranging from benign to fatal. Early recognition by abdominal CT scan and prompt treatment are essential to improve survival. 
